# High Dephosphorylated-Uncarboxylated MGP in Hemodialysis patients: risk factors and response to vitamin K_2_, A pre-post intervention clinical trial

**DOI:** 10.1186/s12882-017-0609-3

**Published:** 2017-06-07

**Authors:** Mabel Aoun, Maha Makki, Hiba Azar, Hiam Matta, Dania Nehme Chelala

**Affiliations:** 1 Nephrology Department, Saint-Georges Hospital, Ajaltoun, Lebanon; 2 Nephrology Department, Saint-Joseph University, Beirut, Lebanon; 3 Biostatistics Unit, Clinical Research Institute, American University of Beirut Medical Center, Beirut, Lebanon; 4 Nephrology Department, Hôtel Dieu de France, Beirut, Saint-Joseph University, Beirut, Lebanon; 5 Laboratory Division, Saint-Georges Hospital, Ajaltoun, Lebanon; 6 Head of the Nephrology Department, Hôtel Dieu de France, Beirut. Saint-Joseph University, Beirut, Lebanon

**Keywords:** Dephosphorylated Uncarboxylated matrix Gla protein, Pre-post intervention clinical trial, Hemodialysis, Menaquinone-7, Vascular calcifications, Vitamin K_2_

## Abstract

**Background:**

Vascular calcifications are highly prevalent in hemodialysis patients. Dephosphorylated-uncarboxylated MGP (dp-ucMGP) was found to increase in vitamin K-deficient patients and may be associated with vascular calcifications. Supplementation of hemodialysis patients with vitamin K_2_ (menaquinone-7) has been studied in Europe with a maximum 61% drop of dp-ucMGP levels. The aim of this study is to assess first the drop of dp-ucMGP in an Eastern Mediterranean cohort after vitamin K_2_ treatment and second the correlation between baseline dp-ucMGP and vascular calcification score.

**Methods:**

This is a prospective, pre-post intervention clinical trial involving 50 hemodialysis patients who received daily 360 μg of menaquinone-7 for 4 weeks. At baseline they were assessed for plasma dp-ucMGP levels and vascular calcification scores (AC-24) as well as for other demographic, clinical and biological variables. Dp-ucMGP levels were measured a second time at 4 weeks.

**Results:**

At baseline, dp-ucMGP levels were extremely elevated with a median of 3179.15 (1825.25; 4339.50) pM and correlated significantly with AC-24 (Spearman’s rho = 0.43, *P* = 0.002). Using a bivariate regression analysis, the association between dp-ucMGP levels and AC-24 was most significant when comparing dp-ucMGP levels less than 1000 to those more than 1000 pM (*P* = 0.02). Dp-ucMGP levels higher than 5000 pM were significantly associated with females, patients with recent fracture and patients with lower serum albumin (respectively *P* = 0.02, 0.004 and 0.046).

The average drop of dp-ucMGP at 4 weeks of treatment was found to be 86% with diabetics having the lowest drop rate (*P* = 0.01).

**Conclusion:**

Vitamin K deficiency, as assessed by high dp-ucMGP levels, is profound in hemodialysis patients from the Eastern Mediterranean region and it is significantly correlated with vascular calcifications. Daily 360 μg of menaquinone-7, given for 4 weeks, effectively reduces dp-ucMGP in this population. Future studies are needed to assess the changes in vascular calcifications in hemodialysis patients treated with vitamin K_2_ over a longer follow-up period.

**Trial registration:**

The clinical trial was registered on clinicaltrials.gov (Identification number NCT02876354, on August 11, 2016).

## Background

The majority of patients reaching end-stage renal disease (ESRD) and dialysis suffer from vascular calcifications (VC) and thus are at an increased risk of mortality [1–3]. High scores of VC, even in the absence of cardiovascular risk factors, lead to a higher mortality rate compared to those without calcifications and ≥3 risk factors [4]. In hemodialysis (HD) patients, factors inducing VC are numerous. They include the traditional ones such as age, smoking, diabetes, hypertension, hyperlipidemia and those specific to chronic kidney disease (CKD) such as hyperparathyroidism and hyperphosphatemia. HD patients are therefore at a higher cardiovascular risk and scientists have compelling arguments to look seriously for a reliable screening tool and effective treatment for VC. The 2009 Kidney Disease: Improving Global Outcomes (KDIGO) clinical practice guideline for the Diagnosis, Evaluation, Prevention, and Treatment of Chronic Kidney Disease–Mineral and Bone Disorder (CKD–MBD) has suggested measuring vascular calcifications’ scores by a lateral abdominal radiograph that is a cost-effective alternative to the standard computed tomography-based imaging [5]. In order to lower or prevent VC in CKD patients, many interventions have been studied in the past, such as statins, non-calcium-based phosphate binders and calcimimetics. However none of these interventions proved to have a solid and consistent beneficial effect on VC [6–9].

In the last few years, remarkable data emerged concerning the association between VC and plasmatic levels of dephosphorylated-uncarboxylated MGP (dp-ucMGP) [10–13]. Dp-ucMGP is the precursor of Matrix Gla protein (MGP). MGP is a small protein known to act locally on the arterial wall as a calcification inhibitor and it is vitamin K-dependent [11, 14]. Vitamin K family includes phylloquinone (vitamin K_1_) and several menaquinones (vitamin K_2_). Vitamin K_2_ can be provided to the patient as a pill or through a specific diet and its deficiency leads to a decrease in MGP and an increase in dp-ucMGP. Therefore, supplementing high-risk patients especially CKD patients with vitamin K_2_ seems very promising to slow VC [10, 15, 16]. Menaquinone-7 (MK-7) supplementation was previously studied in healthy individuals and in two hemodialysis European cohorts to define the ideal dose [17–19].

The effect of MK-7 on dp-ucMGP levels has not yet been studied in Eastern Mediterranean populations. With the hypothesis that the Mediterranean diet has its particularities, we conducted a study in a Lebanese hemodialysis center where patients were assigned to receive MK-7 for 4 weeks. The study aimed at assessing first the dp-ucMGP drop after supplementation with MK-7 and second the correlation between dp-ucMGP and the Aortic Calcification Severity (AC-24) score at baseline.

## Methods

### Trial design, participants and data collection

This is a prospective, pre-post intervention clinical trial.

All patients in our center older than 18 years on chronic hemodialysis for more than 1 month were included. Patients who refused to sign the consent form and those who were on vitamin K antagonists (VKA) and could not stop it (those with heart valve replacements) were excluded. The total trial duration was 4 weeks. Demographics, comorbidities, medications and laboratory data were collected from the patients’ medical records. Variables that were collected include: age, gender, body mass index (BMI), dialysis vintage, residual diuresis, diabetes, hypertension, smoking, coronary artery disease (CAD), previous parathyroidectomy (PTHX), recent fracture (in the last 6 months), calcium and non-calcium-based phosphate binders (NCBB) use and daily dose, cinacalcet use, alfacalcidol weekly dose, AC-24 score, dp-ucMGP level, LDL cholesterol, urea reduction ratio (URR), serum albumin, means of serum calcium, serum phosphorus and second generation intact parathyroid hormone (PTH). Serum calcium and phosphorus are measured in our center on a monthly basis: the mean of the last 12 measurements was calculated. Serum PTH is measured every 4–6 months: we collected all PTH levels of each patient since the start of dialysis in order to obtain the mean PTH level.

### Definitions

Coronary artery disease (CAD) was defined as a history of coronary artery disease, documented by cardiac catheterization and treated medically, by PTCA or CABG. Patients were considered diabetics or hypertensive if taking antidiabetic or antihypertensive treatment respectively. Non-calcium-based phosphate binders available in our center were sevelamer hydrochloride and lanthanum carbonate. The only active vitamin D that our patients were taking was alfacalcidol. Normal serum phosphate and calcium levels were defined respectively as 3.5–4.5 mg/dL and 8.4–9.5 mg/dL and vitamin K deficiency as dp-ucMGP level > 500 pM.

### Ethical considerations

The study was approved by the ethics committee of the Saint-Joseph University-Beirut (approval number HDF-861). It complies with the Declaration of Helsinki of 1975. All patients included in the study gave their written informed consent. The clinical trial was registered on clinicaltrials.gov (Identification number NCT02876354).

### Intervention

All patients received 360 μg of MK-7 once daily (2 capsules of 180 μg). MK-7 was provided by Omicron Pharmaceuticals, Lebanon. The chosen dose in this trial was established according to two previous trials conducted in hemodialysis patients in Europe [17, 18]. In order to ensure the compliance with the drug, the dose on the day of dialysis was given post-dialysis in the center. As for the dose on the day without dialysis, patients were advised to take it with lunch. Besides, the medication boxes were checked and remaining pills counted at the end of the study. Before starting the trial, patients on VKA as a preventive measure for vascular access thrombosis were asked to stop it and take clopidogrel instead; the levels of dp-ucMGP were then measured weekly until their stabilization. They were then assigned to receive 360 μg of MK-7 once daily as the rest of the patients. We assessed the average time necessary to reach a stable level of dp-ucMGP after VKA withdrawal.

### Analysis of dp-ucMGP and AC-24 score measurements

Dp-ucMGP level was measured twice, at baseline before the vitamin K_2_ supplementation and after 4 weeks of treatment. Pre-dialysis venous blood samples were processed at the same laboratory. Circulating plasma dp-ucMGP has been quantified using a dual antibody enzyme-linked immunosorbent assay provided by Immunodiagnostic Systems Ltd., United Kingdom.

Lateral abdominal X-ray of the lumbar aorta was performed at the initiation of the study. The abdominal aortic calcifications were estimated for each patient using the Aortic Calcification Severity (AC-24) score [20]. Two independent physicians calculated the AC-24 score, one of them was blinded. If the difference was one point, the higher score was recorded. If the difference was more than one point a third opinion was taken and the mean of the three levels was retained.

### Adverse events and safety

Patients were evaluated at each dialysis session for any side effect especially gastrointestinal intolerance of the medication, vascular access patency or any other thrombotic event (assessed by fistula auscultation and legs’ examination).

### Statistical analysis

Descriptive statistics were summarized by presenting the number and percentage for categorical variables and median and interquartile range (IQR) for continuous variables. The association between different dp-ucMGP levels and other categorical variables was carried out by using the Fisher’s exact test. Mann-Whitney U test was used for the association with continuous variables. Multivariate regression analysis was used to adjust for potentially confounding variables. The logistic regression analysis assessed the association between the different groups of dp-ucMGP level drop (≤90% versus >90%) and the different predictors. The correlation between two continuous variables was assessed by using scatter plots and Spearman’s rank correlation coefficient. *P*-value <0.05 was used to indicate statistical significance. All statistical analyses were performed using the Statistical Package for Social Sciences (SPSS, version 24).

### Outcome measures

The primary endpoint was the percentage of dp-ucMGP drop after 4 weeks of vitamin K_2_ supplementation at a dose of 360 μg /d. Secondary endpoints were: 1- the correlation between AC-24 score and baseline dp-ucMGP levels, 2- the correlation between baseline dp-ucMGP levels and the other collected variables, 3- the correlation between dp-ucMGP drop and the other collected variables.

## Results

### Baseline demographics and clinical characteristics:

**Fig. 1:**
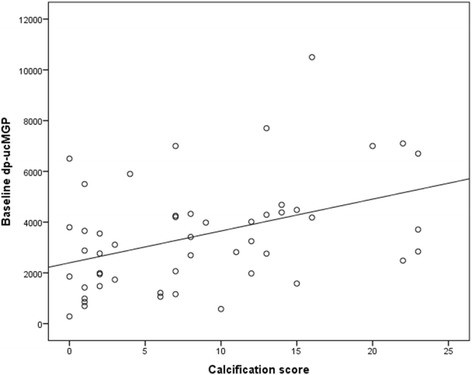
Scatter plot for the association between calcification score (AC-24) and dp-ucMGP level (pM) at baseline (Spearman correlation coefficient = 0.43, *p* = 0.002)

Fifty patients were included in the trial (Table 1). They all received 360 μg of MK-7 once daily. The median age was 71.50 (56.75; 79.25) years. 60% were males. 36% were diabetics. Median BMI was 25.82 (22.85; 28.42) Kg/m^2^. 4% were on cinacalcet and 40% on non-calcium-based phosphate binders. Their median time on dialysis was 47.0 (26.5; 110.5) months. Median PTH was 188.50 (117.75; 266.50) pg/ml. Median serum albumin was 39.0 (36.0; 41.0) g/l. 28% had documented CAD. All patients with a recent fracture had dp-ucMGP levels >5000 pM. Median AC24 score of all patients was 7.0 (2.0; 13.25). 98% had increased dp-ucMGP at baseline (>500 pM). Using a univariate regression analysis, a strong correlation was demonstrated between baseline dp-ucMGP and calcification score (*P* = 0.002) (Fig. 1).

**Table 1:** Baseline characteristics of the 50 patients

	ALL *N* = 50
Age (years), median (IQR)	71.50 (56.75; 79.25)
Gender, male n (%)	30 (60.0)
Dialysis vintage (months), median (IQR)	47.0 (26.5; 110.5)
Diabetes, *n* (%)	18 (36.0)
Hypertension, *n* (%)	38 (76.0)
Smoking, *n* (%)	8 (16.0)
Calcification score, median (IQR)	7.0 (2.0; 13.25)
Baseline dp-ucMGP (pM), median (IQR)	3179.15 (1825.25; 4339.50)
NCBB, *n* (%)	20 (40.0)
Residual diuresis, *n* (%)	6 (12.0)
Calcium dose (tablets of 600 mg), median (IQR)	1.00 (1.00; 2.25)
Alfacalcidol dose (μg), median (IQR)	0.75 (0.00; 1.50)
Serum phosphate (mg/dl), median (IQR)	3.90 (3.17; 4.65)
Serum calcium (mg/dl), median (IQR)	9.10 (8.80; 9.30)
PTH (pg/ml), median (IQR)	188.50 (117.75; 266.50)
LDL cholesterol (mg/dl), median (IQR)	92.0 (77.0; 113.0)
BMI (Kg/m^2^), median (IQR)	25.82 (22.85; 28.42)
URR, median (IQR)	75.0 (71.0; 79.0)
Albumin (g/l), median (IQR)	39.0 (36.0; 41.0)
CAD, *n* (%)	14 (28.0)
Cinacalcet, *n* (%)	2 (4.0)
Previous acenocoumadin, n (%)	6 (12.0)
PTHX, *n* (%)	2 (4.0)
Recent fracture, *n* (%)	3 (6.0)

Abbreviations: *NCBB* non-calcium-based phosphate binders, *PTH* intact parathyroid hormone, *BMI* body mass index, *URR* urea reduction ratio, *CAD* documented coronary artery disease, *PTHX* previous parathyroidectomy

Six patients were on VKA before their inclusion in the trial and were switched to clopidogrel 1 month before. All of them had a baseline dp-ucMGP >5000 pM. The percentage of decrease of dp-ucMGP levels after 2 weeks of VKA withdrawal was 70%. The indication for VKA therapy in those patients was vascular access patency. After dropping VKA and treating them for 4 weeks by vitamin K_2_ no thrombotic events were noted.

### Bivariate regression analysis between different groups of dp-ucMGP levels (Table 2, Table 3, Table 4)

When patients with dp-ucMGP <1000 were compared to those >1000 pM, older age and higher calcification scores were found to be associated with levels >1000 pM (*P* = 0.02). When a median cut-off of 3000 pM was used, only high calcification score was significantly associated with levels >3000 pM (*P* = 0.04). And when we compared patients with a dp-ucMGP baseline level < 5000 to those >5000 pM, females, patients with a recent fracture and patients with lower serum albumin levels displayed the higher levels of dp-ucMGP (respectively *P* = 0.02, 0.004 and 0.046).

**Table 2:** Bivariate regression analysis between patients with baseline dp-ucMGP <5000 and those >5000 pM

	Baseline dp-ucMGP <5000 *N* = 41	Baseline dp-ucMGP >5000 *N* = 9	*P*-value
Age (years), median (IQR)	69.0 (56.5; 76.5)	80.0 (57.0; 82.5)	0.18
Gender, male *n* (%)	28 (68.3)	2 (22.2)	0.02
Dialysis vintage (months), median (IQR)	43.0 (20.5; 104.0)	82.0 (41.0; 134.0)	0.18
Diabetes, *n* (%)	16 (39.0)	2 (22.2)	0.46
Hypertension, *n* (%)	34 (82.9)	4 (44.4)	0.03
Smoking, *n* (%)	6 (14.6)	2 (22.2)	0.62
Calcification score, median (IQR)	7.0 (2.0; 12.5)	13.0 (2.5; 21.0)	0.25
NCBB, *n* (%)	16 (39.0)	4 (44.4)	1.00
Residual diuresis, *n* (%)	5 (12.2)	1 (11.1)	1.00
Calcium dose (tablets of 600 mg), median (IQR)	1.0 (1.0; 3.0)	1.0 (0.0; 1.0)	0.055
Alfacalcidol dose (μg), median (IQR)	0.75 (0.00; 1.25)	1.0 (0.5; 5.0)	0.08
Serum phosphate (mg/dl), median (IQR)	3.9 (3.3; 4.8)	3.90 (2.75; 4.40)	0.29
Serum calcium (mg/dl), median (IQR)	9.1 (8.8; 9.3)	9.2 (8.8; 9.6)	0.40
PTH (pg/ml), median (IQR)	163.0 (95.2; 260.0)	197.0 (165.5; 397.0)	0.27
LDL cholesterol (mg/dl), median (IQR)	92.0 (77.0; 113.0)	92.0 (67.0; 113.0)	0.82
BMI, median (IQR)	25.6 (22.8; 27.8)	26.16 (23.35; 33.56)	0.18
URR, median (IQR)	74.0 (71.0; 78.0)	77.0 (75.0; 80.5)	0.08
Albumin, median (IQR)	39.0 (37.0; 41.5)	36.5 (34.0; 39.0)	0.046
CAD, *n* (%)	11 (26.8)	3 (33.3)	0.70
Cinacalcet, *n* (%)	1 (2.4)	1 (11.1)	0.33
Previous acenocoumadin, *n* (%)	4 (9.8)	2 (22.2)	0.29
PTHX, *n* (%)	2 (4.9)	0 (0.0)	1.00
Recent fracture, *n* (%)	0 (0.0)	3 (33.3)	0.004

Abbreviations: *NCBB* non-calcium-based phosphate binders, *PTH* intact parathyroid hormone, *BMI* body mass index, *URR* urea reduction ratio, *CAD* documented coronary artery disease, *PTHX* previous parathyroidectomy

**Table 3:** Bivariate regression analysis between patients with baseline dp-ucMGP <1000 and those >1000 pM

	Baseline dp-ucMGP <1000 *N* = 5	Baseline dp-ucMGP >1000 *N* = 45	*P*-value
Age (years), median (IQR)	55.0 (38.0; 64.0)	73.0 (61.0; 80.0)	0.02
Gender, male *n* (%)	3 (60.0)	27 (60.0)	1.00
Dialysis vintage (months), median (IQR)	36.0 (9.5; 152.0)	47.0 (28.5; 108.5)	0.73
Diabetes, *n* (%)	2 (40.0)	16 (35.6)	1.00
Hypertension, *n* (%)	3 (60.0)	35 (77.8)	0.58
Smoking, *n* (%)	0 (0.0)	8 (17.8)	0.58
Calcification score, median (IQR)	1.0 (0.5; 5.5)	8.0 (2.0; 14.0)	0.02
NCBB, *n* (%)	2 (40.0)	18 (40.0)	1.00
Residual diuresis, *n* (%)	0 (0.0)	6 (13.3)	1.00
Calcium dose (tablets of 600 mg), median (IQR)	3.0 (1.5; 3.0)	1.0 (1.0; 2.0)	0.04
Alfacalcidol dose (μg), median (IQR)	0.75 (0.25; 1.87)	0.75 (0.00; 1.50)	0.90
Serum phosphate (mg/dl), median (IQR)	3.9 (3.6; 6.6)	3.9 (3.0; 4.6)	0.29
Serum calcium (mg/dl), median (IQR)	9.3 (9.05; 9.65)	9.1 (8.8; 9.2)	0.09
PTH (pg/ml), median (IQR)	127.0 (82.5; 297.0)	196.0 (118.0; 270.0)	0.29
LDL cholesterol (mg/dl), median (IQR)	80.0 (72.0; 113.0)	93.0 (77.0; 113.0)	0.59
BMI (Kg/m^2^), median (IQR)	30.2 (17.9; 34.3)	25.8 (22.9; 27.8)	0.69
URR, median (IQR)	71.0 (65.0; 81.5)	75.0 (72.0; 79.0)	0.46
Albumin, median (IQR)	40.0 (35.5; 44.5)	39.0 (36.0; 41.0)	0.44
CAD, *n* (%)	1 (20.0)	13 (28.9)	1.00
Cinacalcet, *n* (%)	0 (0.0)	2 (4.4)	1.00
Previous acenocoumadin, *n* (%)	2 (40.0)	4 (8.9)	0.10
PTHX, *n* (%)	0 (0.0)	2 (4.4)	1.00
Recent fracture, *n* (%)	0 (0.0)	3 (6.7)	1.00

Abbreviations: *NCBB* non-calcium-based phosphate binders, *PTH* intact parathyroid hormone, *BMI* body mass index, *URR* urea reduction ratio, *CAD* documented coronary artery disease, *PTHX* previous parathyroidectomy

**Table 4:** Bivariate regression analysis between patients with baseline dp-ucMGP <3000 and those >3000 pM

	Baseline dp-ucMGP <3000 *N* = 24	Baseline dp-ucMGP >3000 *N* = 26	*P*-value
Age (years), median (IQR)	69.0 (53.5; 76.5)	73.5 (61.7; 80.0)	0.28
Gender, male *n* (%)	17 (70.8)	13 (50.0)	0.16
Dialysis vintage (months), median (IQR)	40.0 (17.0; 99.2)	49.5 (37.7; 119.0)	0.15
Diabetes, *n* (%)	11 (45.8)	7 (26.9)	0.24
Hypertension, *n* (%)	18 (75.0)	20 (76.9)	1.00
Smoking, *n* (%)	3 (12.5)	5 (19.2)	0.70
Calcification score, median (IQR)	4.5 (1.0; 10.7)	10.5 (3.7; 15.2)	0.04
NCBB, *n* (%)	11 (45.8)	9 (34.6)	0.56
Residual diuresis, *n* (%)	4 (16.7)	2 (7.7)	0.41
Calcium dose (tablets of 600 mg), median (IQR)	2.0 (1.0; 3.0)	1.0 (1.0; 2.0)	0.10
Alfacalcidol dose (μg), median (IQR)	0.75 (0.00; 1.50)	0.75 (0.25; 1.50)	0.69
Serum phosphate (mg/dl), median (IQR)	3.85 (3.50; 5.07)	3.9 (3.0; 4.5)	0.20
Serum calcium (mg/dl), median (IQR)	9.0 (8.7; 9.3)	9.15 (8.90; 9.32)	0.13
PTH (pg/ml), median (IQR)	158.5 (97.1; 260.2)	205.0 (126.0; 319.0)	0.38
LDL cholesterol (mg/dl), median (IQR)	93.5 (77.7; 115.0)	92.0 (76.0; 113.0)	0.56
BMI (Kg/m^2^), median (IQR)	25.57 (22.74; 29.64)	26.02 (22.76; 28.42)	0.74
URR, median (IQR)	75.0 (69.5; 78.7)	75.00 (71.25; 79.25)	0.76
Albumin, median (IQR)	39.0 (35.2; 41.0)	38.50 (36.00; 41.25)	0.99
CAD, *n* (%)	6 (25.0)	8 (30.8)	0.76
Cinacalcet, *n* (%)	1 (4.2)	1 (3.8)	1.00
Previous acenocoumadin, *n* (%)	3 (12.5)	3 (11.5)	1.00
PTHX, *n* (%)	0 (0.0)	2 (7.7)	0.49
Recent fracture, *n* (%)	0 (0.0)	3 (11.5)	0.24

Abbreviations: *NCBB* non-calcium-based phosphate binders, *PTH* intact parathyroid hormone, *BMI* body mass index, *URR* urea reduction ratio, *CAD* documented coronary artery disease, *PTHX* previous parathyroidectomy

### Drop of dp-ucMGP after 4 weeks of vitamin K_2_ supplementation

After 4 weeks of vitamin K2 supplementation, the median level of dp-ucMGP decreased from 3179.15 (1825.25; 4339.50) to 294.50 (217.02; 381.55) pM. This translates into an 86% drop. Using bivariate and multivariate regression analysis, non-diabetic patients displayed significantly higher drop in dp-ucMGP levels (*P* = 0.01) (Tables 5,6).

**Table 5:** Bivariate regression analysis: variables associated with the dp-ucMGP drop divided by the median (90%)

	Dp-ucMGP drop ≤90% *N* = 23	Dp-ucMGP drop >90% *N* = 27	*P-value*	*OR (95% CI)*
Age (years), Median (IQR)	72.0 (55.0; 80.0)	69.0 (63.0; 79.0)	0.63	1.01 (0.97–1.05)
Gender, male *n* (%)	14 (60)	16 (59.3)	1.00	1.07 (0.34–3.33)
Dialysis vintage (months), Median (IQR)	41.0 (28.0; 112.0)	48.0 (22.0; 110.0)	0.68	1.02 (0.92–1.14)
Diabetes, *n* (%)	13 (56.5)	5 (18.5)	0.01	0.17 (0.05–0.62)
Hypertension, *n* (%)	16 (69.6)	22 (81.5)	0.32	1.92 (0.52–7.18)
Smoking, *n* (%)	3 (13.0)	5 (18.5)	0.71	1.51 (0.32–7.17)
Calcification score, Median (IQR)	3.0 (1.0; 12.0)	9.0 (4.0; 14.0)	0.10	1.06 (0.97–1.15)
NCBB, *n* (%)	10 (43.5)	10 (37.0)	0.64	0.76 (0.25–2.38)
Residual diuresis, *n* (%)	3 (13.0)	3 (11.1)	1.00	0.83 (0.15–4.59)
Calcium dose, Median (IQR)	2.0 (1.0; 3.0)	1.0 (1.0; 2.0)	0.12	0.63 (0.36–1.11)
Alfacalcidol dose (μg), Median (IQR)	0.75 (0.00; 1.50)	0.75 (0.25; 1.50)	0.20	1.17 (0.82–1.69)
Serum phosphate (mg/dl), Median (IQR)	3.90 (3.50; 5.00)	3.90 (3.00; 4.50)	0.25	0.64 (0.39–1.06)
Serum calcium (mg/dl), Median (IQR)	9.00 (8.70; 9.30)	9.20 (8.90; 9.30)	0.08	4.97 (0.82–30.06)
PTH (pg/ml), Median (IQR)	153.00 (88.50; 216.00)	216.0 (144.0; 314.0)	0.10	1.03 (0.98–1.07)
LDL cholesterol (mg/dl), Median (IQR)	93.50 (77.75; 114.25)	92.00 (76.00; 113.25)	0.61	0.99 (0.97–1.02
BMI (Kg/m^2^), Median (IQR)	26.45 (23.03; 31.39)	25.26 (22.65; 26.65)	0.13	0.89 (0.78–1.01)
URR, Median (IQR)	75.0 (71.0; 79.0)	74.0 (71.0; 79.0)	0.70	0.98 (0.88–1.10)
Albumin, Median (IQR)	37.0 (35.0; 40.0)	39.0 (37.0; 41.0)	0.15	1.09 (0.94–1.26)
CAD, *n* (%)	7 (30.4)	7 (25.9)	0.72	0.80 (0.23–2.76)
Cinacalcet, *n* (%)	1 (4.3)	1 (3.7)	1.00	0.85 (0.05–14.33)
Previous acenocoumadin, *n* (%)	3 (13.0)	3 (11.1)	1.00	0.83 (0.15–4.59)
PTHX, *n* (%)	0 (0.0)	2 (7.4)	0.49	NA
Recent fracture, *n* (%)	1 (4.3)	2 (7.4)	1.00	1.76 (0.15–20.76)

Abbreviations: *NCBB* non-calcium-based phosphate binders, *PTH* intact parathyroid hormone, *BMI* body mass index, *URR* urea reduction ratio, *CAD* documented coronary artery disease, *PTHX*, previous parathyroidectomy

**Table 6:** Multivariate logistic regression analysis of potential predictors of drop of dp-ucMGP

Drop MGP (reference: ≤90%)		
Variables	OR (95% CI)	*P*-value
Diabetes	0.17 (0.05–0.62)	0.01

Variables included in the model were:

Sex, age, BMI, calcification score, dialysis vintage, NCPB, alfacalcidol dose, calcium dose, cinacalcet, previous acenocoumadin, serum albumin, serum calcium, serum phosphate, PTH, smoking, diabetes, hypertension, LDL Cholesterol, URR, CAD, recent fracture.

**Fig. 2:**
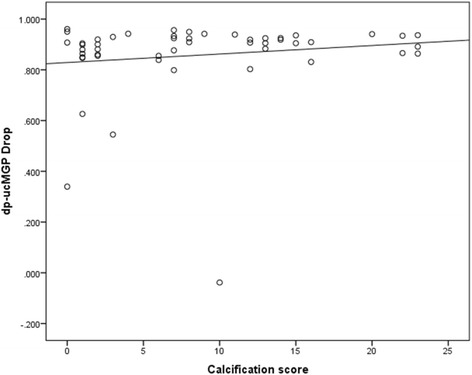
Scatter plot for the association between aortic calcification score (AC-24) and dp-ucMGP drop (Spearman correlation coefficient = 0.19, *p =* 0.18)

88% of all patients reduced their dp-ucMGP to less than 500 pM after 4 weeks of treatment. Reaching very low levels was more evident in females and those with a recent fracture (*P* = 0.03 in both). There was no correlation between baseline vascular calcification scores (AC-24) and the dp-ucMGP drop (Fig. 2).

### Adverse events and safety

No thrombotic events were found on physical exam during the 4 weeks of treatment in all 50 patients and no other side effect or complaint was noted. The medication was well tolerated and there was no withdrawal of treatment.

## Discussion

This is the first study to show that hemodialysis patients in the Eastern Mediterranean region have very high levels of dp-ucMGP. It denotes profound vitamin K deficiency and concurs well with previous European findings [17, 18]. It also supports the concept that HD patients have low vitamin K_2_ intake that was estimated to be 40% less than healthy individuals [21]. Moreover, our findings confirmed that dp-ucMGP >1000 compared to <1000 pM in HD patients is significantly associated with age. Indeed, several reports in the literature have revealed that dp-ucMGP levels increase gradually after the age of 40 and are significantly higher in individuals older than 65 [22, 23]. Liabeuf et al. have shown a significant association with BMI and intact PTH when comparing dp-ucMGP >559.5 to <559.5 pM in diabetics. This was not found in our hemodialysis patients where baseline levels of dp-ucMGP were very high [23]. Regarding dialysis vintage, our patients with longer duration on hemodialysis tended to have higher levels of dp-ucMGP but the association was not statistically significant (*P* = 0.18). This association was not consistent in the literature. Caluwe et al. have demonstrated a significant association between dp-ucMGP and dialysis vintage [17] whereas Schlieper et al. have not [24]. This needs further assessment by larger studies.

Remarkably, our study revealed that dp-ucMGP increases linearly with the increase of the calcification score assessed by a lateral abdominal X-ray (AC-24). This correlation between high dp-ucMGP and calcification scores was not found by Schlieper et al. [24] and not searched for by Caluwe et al. [17] however it is in good agreement with the findings of Delanaye et al. that showed a significant correlation between dp-ucMGP and VC assessed also by AC24 score [13]. Our two studies suggest that a lateral abdominal X-ray and a level of dp-ucMGP are both adequate tools to evaluate VC in hemodialysis patients. It is noteworthy that dp-ucMGP has been proposed as a marker of cardiovascular risk in advanced chronic kidney disease [25]. This could also apply to hemodialysis patients.

Another common finding between Delanaye et al. and our study is the association between low serum albumin level and high dp-ucMGP, emphasizing the importance of malnutrition as a risk factor for high dp-ucMGP as it is a traditional risk factor for high mortality. Interestingly, our cohort is the first to highlight a significant difference between genders when it comes to vitamin K status. Female patients in our center seem to have lower vitamin K_2_ intake and consequently higher dp-ucMGP >5000 pM. It would be interesting in the future to study the intake and absorption of vitamin K_2_ between females in different countries and even subnational groups. Our stratification of dp-ucMGP based on three cut-offs showed also that associated factors with dp-ucMGP can change with the level ranges: in levels < 3000 pM, the most important associated factor is vascular calcification and in very high dp-ucMGP >5000 pM, the predominant factors are those related to poor nutrition (serum albumin and probably female gender).

Most importantly, we demonstrated that daily supplementation of HD patients with 360 μg of MK-7 decreases dp-ucMGP levels by 86% after 4 weeks. This percentage differs considerably from previous European cohorts [17, 18, 24]. Westenfeld et al. treated 14 hemodialysis patients with 360 μg of MK-7 daily and showed a reduction rate of dp-ucMGP by 61.1% after 6 weeks of treatment. Their mean baseline of dp-ucMGP was 2930 pM which is very similar to the mean baseline level of our patients [18]. Caluwé et al. demonstrated a reduction rate of dp-ucMGP levels of 17%, 33% and 46% after 8 weeks of treatment with vitamin K_2_ at the thrice-weekly dose of 360 μg, 720 μg and 1080 μg respectively [17]. The difference in the drop rate of dp-ucMGP between previous studies and ours is not completely understood. It might be explained by the variability of absorption between individuals and ethnicities. The drop of dp-ucMGP was significantly more pronounced in our non-diabetic patients pointing out to the probable poor drug absorption by diabetics. Vitamin K_2_ is also a fat-soluble vitamin, better absorbed with food and our patients received their dose of non-dialysis days with lunch; on the contrary MK-7 in the previous studies was given after dialysis sessions and probably distant from food intake. The drug’s formulation, whether a capsule or a tablet, may also play a role. We used a capsule form in our patients and it was better tolerated. On the contrary, the tablets had a strong smell and led to some withdrawals in the study of Caluwe et al.

This work helped also in assessing the time necessary to wash out the effect of VKA on dp-ucMGP. VKAs are increasingly incriminated in VC and even calciphylaxis [26]. Thus many experts agreed lately on avoiding VKA treatment in dialysis patients [27, 28]. In our study, after 2 weeks of VKA withdrawal, patients reached a stable level of dp-ucMGP. In addition, switching patients from VKA to clopidogrel and supplementing them with vitamin K_2_ did not put them at risk of vascular access thrombosis. This supports the concept of safety of vitamin K_2_ supplementation that was studied in a prospective cohort of 35,000 healthy subjects [29].

We admit two limitations in this study; the first is the small sample size and the second is the absence of a control group. However the good response of all patients to vitamin K_2_ allows us to conclude that a capsule of 360 μg of MK-7 is effective and safe to treat vitamin K deficiency in hemodialysis patients.

## Conclusion

Hemodialysis patients have profound vitamin K deficiency as assessed by high dp-ucMGP plasma levels. High dp-ucMGP level is significantly correlated with high aortic calcification scores and thus can be used as a non-invasive marker for vascular calcifications. The daily administration of 360 μg of vitamin K_2_ (MK-7) decreased dp-ucMGP by 86% after 4 weeks and it was well tolerated. Further studies should be conducted to assess the change in vascular calcifications after an extended duration of therapy.

## Abbreviations

AC-24: Aortic calcification score
BMI: Body mass index
CAD: Coronary artery disease
CKD: Chronic kidney disease
Dp-ucMGP: Dephosphorylated uncarboxylated matrix gla protein
ESRD: End-stage renal disease
HD: Hemodialysis
MK-7: Menaquinone-7
NCBB: Non-calcium-based phosphate binders
PTH: Parathyroid hormone
PTHX: Parathyroidectomy
URR: Urea reduction ratio
VC: Vascular calcifications
